# Expanding Hybrid Studies for Implementation Research: Intervention, Implementation Strategy, and Context

**DOI:** 10.3389/fpubh.2019.00325

**Published:** 2019-11-08

**Authors:** Christopher G. Kemp, Bradley H. Wagenaar, Emily E. Haroz

**Affiliations:** ^1^Department of Global Health, University of Washington, Seattle, WA, United States; ^2^Department of Epidemiology, University of Washington, Seattle, WA, United States; ^3^Department of International Health, Johns Hopkins University, Baltimore, MD, United States; ^4^Center for American Indian Health, Johns Hopkins University, Baltimore, MD, United States

**Keywords:** hybrid studies, intervention, implementation strategy, context, implementation science

## Abstract

Successful implementation reflects the interplay between intervention, implementation strategy, and context. Hybrid effectiveness-implementation studies allow investigators to assess the effects of both intervention and implementation strategy, though the role of context as a third independent variable (IV) is incompletely specified. Our objective is to expand the hybrid typology to include mixtures of all three types of IVs: intervention, implementation strategy, and context. We propose to use **I** to represent the IV of intervention, **IS** to represent implementation strategy, and **C** to represent context. Primary IVs are written first and in upper case. Secondary IVs are written after a forward slash and in lower case; co-primary IVs are written after a dash and in upper case. The expanded framework specifies nine two-variable hybrid types: **I/is**, **I-IS**, **IS/i**, **IS/c**, **IS-C**, **C/is**, **C/i**, **I-C**, and **I/c**. We describe four in detail: **I/is**, **IS/c**, **IS-C**, and **C/is**. We also specify seven three-variable hybrid types. We argue that many studies already meet our definitions of two- or three-variable hybrids. Our proposal builds from the typology proposed by Curran et al. (1), but offers a more complete specification of hybrid study types. We need studies that measure the implementation-related effects of variations in contextual determinants, both to advance the science and to optimize intervention delivery in the real world. Prototypical implementation studies that evaluate the effectiveness of an implementation strategy, in isolation from its context, risk perpetuating the gap between evidence and practice, as they will not generate context-specific knowledge around implementation, scale-up, and de-implementation.

## Introduction

Successful implementation reflects the complex interplay between intervention, implementation strategy, and context (2). Of these, predominant typologies of implementation research emphasize the *implementation strategy*—a specific approach or combination of approaches to facilitate, strengthen, and/or sustain the delivery of interventions in real-world settings (3, 4)—as the *independent variable* of interest to the field (5–7). Prototypical implementation studies aim to estimate the effects of implementation strategies on *implementation outcomes* like adoption, penetration, or fidelity of intervention delivery (8, 9). Hybrid effectiveness-implementation studies are increasingly popular and allow investigators to assess *intervention* effects on patient health alongside *implementation strategy* effects on implementation outcomes (1), with the aim of accelerating the translational research pipeline (10). However, the role of *context* as a third potential independent variable in implementation research remains incompletely specified.

Contextual determinants operate at multiple stages and across multiple levels to promote or inhibit intervention and implementation strategy effectiveness (11). Interventions and implementation strategies must be carefully chosen to suit different contexts, and adapted as necessary (12, 13). Contextual determinants may serve as preconditions for intervention and implementation strategy effectiveness, meaning mechanisms of effect will not be activated in their absence. Contextual determinants may act as moderators, interacting with mechanisms of effect to strengthen or weaken the influence of interventions or implementation strategies on relevant outcomes. Finally, contextual determinants may be part of the mechanism of effect, mediating between the intervention or the implementation strategy and the outcomes of interest (14). Implementation researchers might therefore be interested in estimating and understanding the effects of context on implementation process and outcomes, just as intervention researchers might be interested in the effects of context on intervention effectiveness. The importance of this interplay of context with intervention and implementation strategy effectiveness increases as interventions are scaled across diverse settings.

**Figure 1:**
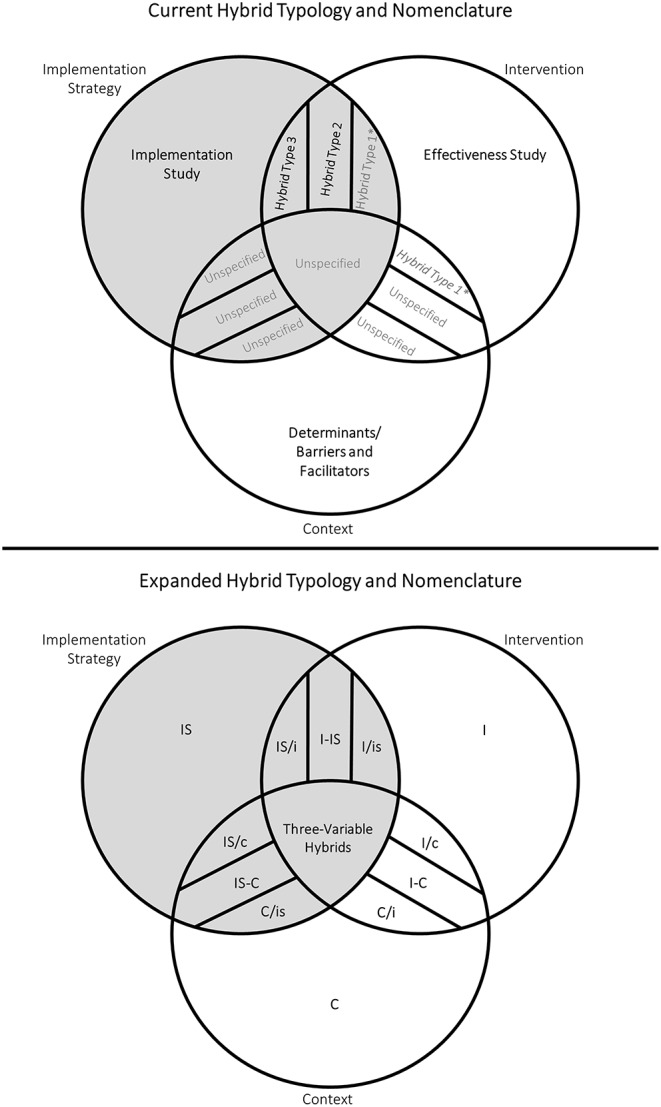
Current and expanded hybrid typologies for implementation research. Gray shading represents study types where the assessment of implementation strategies is a primary or secondary aim. Gray text indicates study types that are currently under- or unspecified. Individual types of three-variable hybrids have been excluded for simplicity. *Curran et al. (1) specify that Hybrid Type 1 studies have a primary aim of testing intervention effectiveness and a secondary aim of gathering information on implementation (e.g., reporting implementation outcomes like feasibility and sustainability) or better understanding the context of implementation (e.g., identifying barriers and facilitators) (9). Therefore, we argue that some Type 1 studies mix the independent variables of intervention and implementation strategy (**I/is**), while others mix the independent variables of intervention and context (**I/c**).

Hybrid studies offer a platform for the combined assessment of two or more kinds of independent variables. In their seminal article, Curran et al. (1) specify three types of effectiveness-implementation hybrid studies (Figure 1) (1). **Type 1** studies mix a primary aim of testing intervention effectiveness with a secondary aim of gathering information on implementation or better understanding the context of implementation. Notably, studies of this type are not currently accepted for publication in the field's leading journal given that their primary aim is to test intervention effectiveness (5). **Type 2** studies have co-primary aims to test intervention effectiveness while simultaneously testing the feasibility or utility of implementation strategies, and **Type 3** studies have a primary aim to test implementation strategy effectiveness while observing clinical outcomes as a secondary aim (1). As specified, these three types leave two important gaps. First, the secondary focus of **Type 1** studies is ambiguous: some **Type 1** studies might assess contextual determinants of implementation success, while others might focus on reporting implementation outcomes associated with a specified implementation strategy. Second, none of these hybrid types allow for a primary aim of estimating and understanding the effects of context on implementation process and outcomes.

Our objective is to expand on the Curran et al. (1) framework to include mixtures of all three types of independent variables that are key for implementation research: intervention, implementation strategy, and context. In light of a growing recognition of the importance of context on both intervention effectiveness and implementation strategy effectiveness (15), this expansion of hybrid types will: (1) provide a common language inclusive of current definitions of implementation research; (2) help prioritize neglected research questions; and (3) inform the choice of study designs to answer priority questions.

## Expanded Hybrid Typology

Our expanded typology builds naturally on the idea of hybrid effectiveness-implementation studies by allowing for hybrids of any combination of the three independent variables (IVs): *intervention, implementation strategy*, and *context* (1). We define intervention as any of the seven “Ps”: programs, practices, principles, procedures, products, pills, or policies intended to improve the health of individuals, groups, or populations (6). We define implementation strategy as a specific approach to facilitate, strengthen, and/or sustain the delivery of interventions in real-world settings (3, 4, 6). We define context as the multi-level and interrelated conditions in which an intervention or implementation strategy is implemented.

We propose to use **I** to represent the IV of intervention, **IS** to represent implementation strategy, and **C** to represent context. Hybrid types are then denoted by combining acronyms for IVs. Primary IVs—those that are intentionally varied within the sample, either naturally, purposively, or experimentally—are written first and in upper case. Secondary IVs—variables whose associated outcomes are observed, but which are not intentionally varied within the sample—are written after a forward slash and in lower case; co-primary IVs are written after a dash and in upper case. For example, Curran et al's. (1) **Type 3** study would be denoted by **IS/i**, indicating the primary aim to study an implementation strategy **(IS)** alongside a secondary aim to observe clinical outcomes associated with an intervention **(i)** (1).

### Overview of Two-Variable Hybrid Types

The expanded typology specifies nine two-variable hybrid studies: **I/is, I-IS, IS/i, IS/c, IS-C, C/is, C/i, I-C**, and **I/c** (Figure 1). Six include the assessment of implementation strategy as primary or secondary aim: **I/is, I-IS, IS/i, IS/c, IS-C**, and **C/is** (Table 1). Two of these—**I-IS** and **IS/i**, previously **Type 2** and **Type 3**, respectively—have been fully specified by Curran et al. (1) and do not warrant further description here (1). **I/is** studies were partially specified by Curran et al. (1) as **Type 1**, though we suggest that **Type 1** is actually an ambiguous combination of **I/c** and **I/is**. We therefore describe four two-variable hybrid types that include the assessment of implementation strategy as a primary or secondary aim: **I/is**, **IS/c**, **IS-C**, and **C/is**. The remaining three two-variable hybrid types (**I/c**, **I-C**, and **C/i**) do not include the assessment of implementation strategy and are therefore not discussed in detail here; see Supplementary Table 1 for descriptions of these hybrid types.

**Table 1:** Two-variable hybrid types that include assessment of implementation strategy.

**Hybrid type**	I/is (Type 1)^*^	**I-IS (Type 2)**	**IS/i (Type 3)**	**IS/c**	**IS-C**	**C/is**
What is being varied?	Intervention	Intervention and implementation strategy	Implementation strategy	Implementation strategy	Implementation strategy and context	Context
Research aims	Primary aim: Evaluate intervention effectiveness	Co-primary aim: Evaluate intervention effectiveness	Primary aim: Evaluate implementation strategy effectiveness	Primary aim: Evaluate implementation strategy effectiveness	Co-primary aim: Evaluate implementation strategy effectiveness	Primary aim: Evaluate effects of context
	Secondary aim: Assess implementation outcomes associated with implementation strategy	Co-primary aim: Evaluate implementation strategy effectiveness	Secondary aim: Assess patient health outcomes associated with intervention	Secondary aim: Better understand the context for implementation	Co-primary aim: Evaluate effects of context	Secondary aim: Assess implementation outcomes associated with implementation strategy
Research questions (examples)	Primary question: Will the intervention improve health outcomes?	Co-primary question: Will the intervention improve health outcomes?	Primary question: Will the implementation strategy improve implementation outcomes?	Primary question: Will the implementation strategy improve implementation outcomes?	Co-primary question: Will the implementation strategy improve implementation outcomes?	Primary question: Which contextual factors mediate or moderate the effectiveness of the implementation strategy?
	Secondary question: What are the implementation outcomes of the specified implementation strategy?	Co-primary question: Will the implementation strategy improve implementation outcomes?	Secondary question: Are patient health outcomes acceptable?	Secondary question(s): What are the barriers to and facilitators of implementation of the implementation strategy?	Co-primary question: Which contextual factors mediate or moderate the effectiveness of the implementation strategy?	Secondary question: What are the implementation outcomes of the specified implementation strategy?
Evaluation methods	Primary aim: Quantitative evaluation of causal effects of intervention	Co-primary aim: Quantitative evaluation of causal effects of intervention	Primary aim: Quantitative evaluation of causal effects of implementation strategy	Primary aim: Quantitative evaluation of causal effects of implementation strategy	Co-primary aim: Quantitative evaluation of causal effects of implementation strategy	Primary aim: Quantitative evaluation of causal effects of context
	Secondary aim: Quantitative, qualitative, or mixed-methods evaluation of implementation outcomes	Co-primary aim: Quantitative evaluation of causal effects of implementation strategy	Secondary aim: Quantitative assessment of patient health outcomes	Secondary aim: Qualitative or mixed-methods process evaluation	Co-primary aim: Quantitative evaluation of causal effects of context	Secondary aim: Quantitative, qualitative, or mixed-methods evaluation of implementation outcomes

* *Curran et al. (1) specify that Hybrid Type 1 studies have a primary aim of testing intervention effectiveness and a secondary aim of gathering information on implementation (e.g., reporting implementation outcomes like feasibility and sustainability) or better understanding the context of implementation (e.g., identifying barriers and facilitators). Therefore, we argue that some Type 1 studies mix the independent variables of intervention and implementation strategy (**I/is**), while others mix the independent variables of intervention and context (**I/c)***.

An **I/is** study would have the primary aim of evaluating intervention effectiveness and the secondary aim of assessing implementation outcomes associated with the implementation strategy in use. The intervention is intentionally varied while outcomes associated with the implementation strategy are observed. As with **I/c** studies, these would require modest refinements to traditional effectiveness studies. For example, **I/is** studies might be accomplished through traditional individual- or cluster-randomized controlled trials. **I/is** studies would distinguish themselves from traditional effectiveness (**I**) studies by including complete specification of the implementation strategies used to introduce and deliver the intervention (3), and by measuring at least one implementation outcome linked to the implementation strategy (8). Potential secondary implementation research questions that could be answered with this hybrid type include: What is the acceptability, feasibility, and/or cost of the implementation strategy? What was the fidelity to the implementation strategy during the intervention effectiveness trial? What other implementation strategies might be promising? How might the implementation strategy be adapted to maximize implementation effectiveness? Data on implementation outcomes could come from several sources at the patient, provider, or organizational levels; these data would also help to explain findings from the effectiveness trial. See Supplementary Table 2 for an example of an **I/is** study.

An **IS/c** study would have the primary aim of evaluating implementation strategy effectiveness and the secondary aim of better understanding the context for implementation. This is analogous to an **I/c** hybrid study, though in this case the implementation strategy is intentionally varied, not the intervention; contextual determinants of implementation process and effectiveness are observed. **IS/c** studies might be accomplished through cluster-randomized controlled trials, though they would distinguish themselves from traditional implementation studies by collecting information on multi-level contextual determinants, likely via process evaluation. Potential primary implementation research questions that could be answered with this hybrid type include: Will the implementation strategy improve implementation outcomes? Secondary questions could include: What are the barriers and facilitators to implementation of the implementation strategy? What modifications to our implementation strategy could be made to maximize implementation in this context or others? Contextual data can also help to explain and frame the results of the implementation study. See Supplementary Table 2 for an example of an **IS/c** study.

An **IS-C** study would have two co-primary aims: evaluate implementation strategy effectiveness and evaluate the effects of context on implementation. Implementation strategy and context are both intentionally varied. In cases where context is under experimental control, this might be achieved through a factorial randomized trial. In cases where context is not under experimental control, block or stratified randomization could be used to balance assignment to implementation strategy conditions across contextual conditions. This hybrid type is motivated by the understanding that effectiveness of an implementation strategy is likely moderated by context—meaning studies in one setting may yield different estimates of effectiveness than studies in another setting. Furthermore, the mechanisms of action for the effectiveness of implementation strategies may change across settings—meaning that different contextual determinants may mediate implementation strategy effectiveness in different settings. Potential implementation research questions that could be answered with this hybrid type include: Will the implementation strategy improve implementation outcomes? Which contextual determinants are preconditions for the success of the implementation strategy? Which contextual determinants moderate or mediate the success of the implementation strategy? See Supplementary Table 2 for an example of an **IS-C** study.

Finally, a **C/is** study would have the primary aim of evaluating the effects of context on implementation and the secondary aim of assessing implementation outcomes associated with the implementation strategy in use. Context is intentionally varied while outcomes associated with the implementation strategy are observed. As with **IS-C** studies, optimal study designs will depend on whether contextual conditions are under experimental control. As with **I/is**, **C/is** studies would specify the implementation strategies in use (3), and measure at least one implementation outcome linked to a specific implementation strategy or package of strategies (8). Potential implementation research questions that could be answered with this hybrid type include: Which contextual determinants moderate or mediate the success of an implementation strategy? What modifications to our implementation strategy could be made to maximize adoption of the implementation strategy in these different contexts? See Supplementary Table 2 for an example of a **C/is** study.

### Recommended Conditions for Use

For all two-variable hybrid types proposed here, we assume that there are minimal risks associated with the intervention, the implementation strategy, and the context. Additional recommended conditions include the following. **I/is** studies should be used when: (1) there is strong face validity for a clinical intervention but its effectiveness has yet to be proven; and (2) the strategies for implementing this clinical intervention are unstudied. **IS/c** are recommended for situations when: (1) there is strong face validity for an implementation strategy, but its effectiveness is not established; and (2) the contextual barriers and facilitators to implementation of the implementation strategy are undefined. **IS-C** would be recommended for conditions when: (1) there is strong face validity for both the implementation strategy and the contexts where the strategy will be most useful, but effectiveness in these contexts has yet to be established. Finally, recommended conditions for **C/is** studies include: (1) there is strong face validity that the context influences the implementation strategy but the effects of context on strategy are not established; and (2) how the implementation strategy works in different contexts is not well-understood.

### Overview of Three-Variable Hybrid Types

The expanded typology specifies seven three-variable hybrid studies. Supplementary Table 1 outlines the key characteristics of all seven: **I/is/c**, **IS/i/c**, **C/i/is**, **I-IS/c**, **I-C/is**, I**S-C/i**, and **I-IS-C**. We have excluded these from Figure 1 for simplicity. The eighth possible combination of the three variables (**i/is/c**) is not discussed as it does not include a primary IV.

The aims and research questions associated with these three-variable hybrid types follow from the two-variable types described above. For example, an **I/is/c** study would have a primary aim of evaluating intervention effectiveness, a co-secondary aim of assessing implementation outcomes associated with the implementation strategy used, and a co-secondary aim of better understanding the context for implementation. This could be a traditional effectiveness study that incorporates a process evaluation and reports implementation outcomes linked to a specified implementation strategy. An **I-IS-C** study would have three co-primary aims: evaluate intervention effectiveness, evaluate implementation strategy effectiveness, and evaluate the effects of context on implementation.

Though three-variable hybrid types are more complex than two-variable types, we argue that many studies—including some currently denoted as hybrid **Type 1, 2, or 3**—already meet the definition of a three-variable hybrid. For instance, many **Type 1** studies test intervention effectiveness, measure implementation outcomes associated with the specified implementation strategy, and assess contextual barriers and facilitators to implementation [e.g., (16)]. We would consider such studies to be three-variable hybrid **I/is/c** type given their dual secondary aims related to collecting information on contextual determinants and implementation strategy. Even paradigmatic non-hybrid studies of the effects of implementation strategies often track patient or population health outcomes to monitor progress and allow presentation of health outcomes to stakeholders, and might therefore be considered two- or three-variable hybrid studies.

## Discussion

We propose an expanded typology of hybrid studies for implementation research that explicitly incorporates mixtures of three IVs: intervention, implementation strategy, and context. This proposal builds from the typology proposed by Curran et al. (1), but offers a more complete specification of hybrids that might be of interest to implementation researchers (1) (Table 2). In particular, this expanded typology re-introduces and emphasizes context as a critical IV—not just as a confounder or nuisance variable, but as a potential precondition, moderator, or mediator of implementation process and outcomes. We need studies that are designed and powered to measure the implementation-related effects of variations in contextual determinants, both to advance the science and to optimize delivery of interventions in the real world. Context is not static and contextual determinants cannot be treated as fixed once measured (2); studies should explicitly consider this dynamism. Our expanded typology offers a common language for implementation scientists designing studies that consider the complex inter-relationship of intervention, implementation strategy, and context.

**Table 2:** Key implications of expanded hybrid typology.

We propose an *expansion* of the Curran et al. (1) typology of hybrid studies for implementation research, alongside a revised *nomenclature*. Our expanded typology recognizes *context* as an independent variable warranting careful study alongside *intervention* and *implementation strategy*. Investigators should consider intentionally varying context in order to identify and explain the *contextual moderators* and *mediators* of intervention and implementation strategy effectiveness. These will help optimize the delivery of interventions and implementation strategies across diverse settings. Investigators should consider rigorous *quasi-experimental* study designs alongside traditional experimental designs to assess the complex and time-varying effects of context.

We believe that the gold standard study design will vary for each hybrid type. Note that we use the phrase *hybrid type* to refer to the combination of IVs of interest in each study, and *study design* to refer to the specific approach to collecting and analyzing data (e.g., observational vs. experimental study, individual vs. cluster randomization, parallel vs. stepped wedge trial). Within each hybrid type, there is room for movement across study designs from observational to experimental and from the production of locally-relevant knowledge to the production of generalizable knowledge (6). Gold standard **IS/i** studies might be cluster-randomized trials, assuming investigators have experimental control over implementation strategies. However, randomized designs may not be possible or desirable in the case of **C/is** studies given that: (1) investigators likely do not have experimental control over the context; (2) the contextual determinants of interest may be numerous, multi-level, and time-varying; and (3) the contextual determinants may be particularly sensitive to bias introduced by investigator control and intensive randomization procedures. For example, investigators could use block or stratified randomization to ensure key contextual determinants are balanced across intervention or implementation strategy study arms. In the case of complex and time-varying contextual determinants, rigorous observational or quasi-experimental (non-randomized) study designs—such as controlled interrupted time-series or regression discontinuity—will be essential. Investigators could use g-computation or instrumental variables to estimate time-varying causal effects and minimize confounding (17, 18).

The under-specification of context as an IV in implementation research is reflected in the fact that over three-fourths of the protocols published in the field's leading journal from 2006 to 2017 have been randomized trials (19). While randomization seeks to eliminate observed and unobserved confounding, enabling the estimation of the average causal effect of one intervention or implementation strategy over another, it also minimizes heterogeneity in effects across populations and contexts. By definition, traditional randomized trials say little about how to optimize the delivery of interventions in the diversity of real-world systems that span practice settings. However, we see implementation science moving toward a new stage in the research pipeline: the translation of effective implementation strategies into routine practice. We need growth and development in the methods and study designs capable of identifying and explaining the multitude of contextual moderators and mediators of implementation strategy effectiveness. These might include mixed-methods approaches, factorial designs, stratified randomization, quasi-experimental and time-series designs, and agent-based and other complex systems modeling (20). However, these designs bring their own challenges, and there is a great need to expand our repertoire of research methodology to address current limitations.

We have highlighted the implementation strategy IV as it is currently the predominant focus in the field. However, we believe that all three IVs are critical to advancing implementation science. Prototypical implementation studies that evaluate the effectiveness of an implementation strategy, in isolation from its context, risk perpetuating the persistent gap between evidence and practice: they do not generate context-specific knowledge around implementation, scale-up, and de-implementation. Therefore, we argue that, at minimum, implementation strategy effectiveness studies should include a secondary aim related to collecting data on contextual determinants. Ideally, many would go further and systematically test variations in context and measure moderation and mediation of implementation strategy effectiveness. Results from such studies would arm future implementers with valuable information about precisely how to choose and tailor interventions and implementation strategies to match—and benefit from—their particular contexts.

We propose an expanded and renamed typology of hybrid studies for implementation research. Our aims were to formally recognize the role of context as a potential IV, to provide a nomenclature for existing studies aiming to identify the contextual moderators and mediators of intervention and implementation strategy effectiveness, and to motivate future growth in this critical area. We believe all implementation studies should consider a hybrid approach and carefully consider whether context may be an IV of interest.

## Author Contributions

All authors contributed equally to the conceptualization and drafting of this manuscript, and approved it for publication.

### Conflict of Interest

The authors declare that the research was conducted in the absence of any commercial or financial relationships that could be construed as a potential conflict of interest.

## Supplementary Material

Supplementary Table 1 Description of data: description of all proposed two-variable and three-variable hybrid study types.

Supplementary Table 2 Description of data: examples of **I/is**, **IS/c**, **IS-C**, and **C/is** hybrid types.
