# Supplementary material for: Expanding Hybrid Studies for Implementation Research: Intervention, Implementation Strategy, and Context
Source: Front Public Health. 2019 Nov 8;7:325. doi: 10.3389/fpubh.2019.00325 (PMC6857476; doi:10.3389/fpubh.2019.00325)
Supplement: Supplementary Table 2 — Description of data: examples of I/is, IS/c, IS-C, and C/is hybrid types. [file Table_2.DOCX]

Supplementary Table 2: Examples of **I/is**, **IS/c**, **IS-C**, and **C/is** hybrid types

| **Hybrid type** | **I/is** | **IS/c** | **IS-C** | **C/is** |
| --- | --- | --- | --- | --- |
| Title | An Evaluation of Trauma Focused Cognitive Behavioral Therapy for Children in Zambia | Systems analysis and improvement to optimize pMTCT (SAIA): a cluster randomized trial | Re-Engage Implementation Trial | Organizational culture and climate profiles: relationships with fidelity to three evidence-based practices for autism in elementary schools |
| Funder | Catholic Relief Services; National Institute of Mental Health, National Institutes of Health, United States | Eunice Kennedy Shriver National Institute of Child Health and Human Development, National Institute of Health, United States | Department of Veterans Affairs, Veterans Health Administration, Health Services Research and Development Service | National Institute of Mental Health, National Institutes of Health, United States |
| Description | This study assesses the effects and describes the implementation of a trauma-focused cognitive behavioral therapy (TF-CBT) intervention to address trauma and stress-related symptoms among orphans and vulnerable children in Zambia [1]. The implementation strategy was using an apprenticeship model to train and supervise lay counselors. Children and adolescents were enrolled and assessed for trauma and shame symptoms pre- and post-intervention. Treatment fidelity was tracked using checklists and case notes to monitor utility of the apprenticeship model as an implementation strategy. | The SAIA trial was a pragmatic cluster randomized trial of an implementation strategy (System Analysis and Improvement Approach; SAIA) to optimize and improve the delivery of the package of routinely-delivered evidence-based clinical interventions for the prevention of mother to child transmission of HIV (pMTCT) in Côte d’Ivoire, Kenya, and Mozambique [2,3].  The SAIA implementation strategy is a health facility-based five-step process which includes: (1) pMTCT cascade analysis; (2) process mapping of bottlenecks: (3) identification of system modifications; (4) assessment of system modifications on pMTCT cascade; and (5) repeat analysis and improvement cycles. The SAIA implementation strategy is implemented by health facility staff in public-sector clinics and relies on routine health information system data for primary intervention outcomes. | The Re-Engage Implementation trial was a four-phase, two-arm, longitudinal, clustered randomized trial designed to compare the effectiveness of a standard implementation strategy (Replicating Effective Programs [REP]), compared to REP enhanced with external facilitation, in terms of their promotion of the uptake of the evidence-based Re-Engage population-based outreach program by Veterans Affairs sites [4].  The REP implementation strategy has three central components: making program materials user friendly, training providers, and providing technical assistance to providers. Enhanced REP includes external facilitators who make weekly calls to providers and offer specific guidance on overcoming barriers to implementation. | This was a study of the implementation of evidence-based practices for youth with autism by elementary schools in the United States [5]. Contextual determinants including organizational culture, climate, and work attitudes were assessed pre-implementation, at the beginning of the school year. Fidelity of implementation was observed mid-implementation. Latent profile modeling was used to test for differences in fidelity among schools depending on culture and climate. Implementation strategies were tracked and specified using the Standards for Reporting Implementation Studies (StaRI) checklist. |
| Primary aim | Assess effects of the TF-CBT intervention on trauma and shame symptoms among child and adolescent participants. | Implementation strategy effectiveness, as measured by penetration and fidelity to an evidence-based based clinical protocol to prevent mother to child transmission of HIV including: (1) the proportion of women screened for HIV during first ANC visit; (2) proportion of HIV infected women receiving antiretroviral (ARV) medications during pregnancy; and (3) proportion of infants born to HIV-infected women screened for HIV at six weeks postpartum. | Comparative implementation strategy effectiveness, as measured by uptake, or the percentage of veterans at each facility whose clinical status had been updated in the Re-Engage web-based clinical registry [6]. Secondary outcomes included successful contacts, veteran re-engagement, all-cause mortality, and healthcare utilization. | Assess effects of contextual determinants including organizational culture, climate, and work attitudes on subsequent implementation fidelity. |
| Secondary/co-primary aim | Secondary aim: Monitor treatment fidelity of lay counselors to understand whether the apprenticeship model of training and supervision is a promising implementation strategy. | Secondary aim: A qualitative evaluation organized around the Consolidated Framework for Implementation research (CFIR) to analyze contextual factors associated with SAIA acceptability, penetration, and adoption by pMTCT staff, as well as factors impacting the quality, feasibility, and sustainability of SAIA’s application [7]. | Co-primary aim: Test whether organizational culture and climate moderated the comparative effectiveness of the two implementation strategies [8]. Veterans Affairs sites were assigned organizational culture and climate scores derived from a national survey of Veterans Affairs employees conducted in 2012. Interactions between culture and climate scores and treatment assignment were used to test hypotheses of moderation by contextual determinants. | Secondary aim: Track and specify the implementation strategies used by each school. |
| Situating within the expanded typology | The primary aim of was to assess the **effects of a clinical intervention** for trauma and stress among orphans and vulnerable children. A secondary aim was to collect information on whether the apprenticeship model of training and supervision is a promising **implementation strategy** by monitoring implementation outcomes including fidelity. | The primary aim of the SAIA trial was to test the SAIA **implementation strategy compared to implementation as usual**. In addition, a secondary aim was to collect information on **context** using qualitative interviews organized around the CFIR framework. No data was collected on patient outcomes for clinical interventions, and clinical intervention effectiveness is not being tested, so the variable of **intervention** is not included. | The primary aim of the Re-Engage implementation trial was to test the **comparative effectiveness of two sequences of implementation strategies** (Immediate vs. Delayed Enhanced REP). The co-primary aim achieved by the secondary analysis was to **test whether the contextual determinants** of organizational culture and climate **moderated** this comparative effectiveness. | The primary aim of was to test the **effects of context** on the process and outcomes of implementation. In addition, a secondary aim was to collect information on **implementation strategies** in use. |

**References**

1. Murray LK, Familiar I, Skavenski S, et al. An evaluation of trauma focused cognitive behavioral therapy for children in Zambia. *Child abuse & neglect.* 2013;37(12):1175-1185.

2. Sherr K, Gimbel S, Rustagi A, et al. Systems analysis and improvement to optimize pMTCT (SAIA): a cluster randomized trial. *Implementation Science.* 2014;9(1):55.

3. Rustagi AS, Gimbel S, Nduati R, et al. Impact of a systems engineering intervention on PMTCT service delivery in Côte d’Ivoire, Kenya, Mozambique: a cluster randomized trial. *Journal of acquired immune deficiency syndromes (1999).* 2016;72(3):e68.

4. Kilbourne AM, Abraham KM, Goodrich DE, et al. Cluster randomized adaptive implementation trial comparing a standard versus enhanced implementation intervention to improve uptake of an effective re-engagement program for patients with serious mental illness. *Implementation Science.* 2013;8(1):136.

5. Williams NJ, Frank HE, Frederick L, et al. Organizational culture and climate profiles: relationships with fidelity to three evidence-based practices for autism in elementary schools. *Implementation Science.* 2019;14(1):15.

6. Kilbourne AM, Almirall D, Goodrich DE, et al. Enhancing outreach for persons with serious mental illness: 12-month results from a cluster randomized trial of an adaptive implementation strategy. *Implementation Science.* 2014;9(1):163.

7. Gimbel S, Rustagi AS, Robinson J, et al. Evaluation of a systems analysis and improvement approach to optimize prevention of mother-to-child transmission of HIV using the consolidated framework for implementation research. *Journal of acquired immune deficiency syndromes (1999).* 2016;72(Suppl 2):S108.

8. Smith SN, Almirall D, Prenovost K, et al. Organizational culture and climate as moderators of enhanced outreach for persons with serious mental illness: results from a cluster-randomized trial of adaptive implementation strategies. *Implementation Science.* 2018;13(1):93.
